# Treatment provision for gambling disorder in Britain: call for an integrated addictions treatment and commissioning model

**DOI:** 10.1192/pb.bp.114.050401

**Published:** 2016-06

**Authors:** Sanju George, Henrietta Bowden-Jones

**Affiliations:** 1 Birmingham and Solihull Mental Health NHS Foundation Trust; 2 National Problem Gambling Clinic

## Abstract

Treatment provision for individuals with gambling problems in Britain is at best inadequate. Here we call for gambling treatment provision to be integrated into mainstream drug and alcohol services, and for its commissioning responsibilities to fall under local public health departments.

Gambling disorder sits alongside more traditional substance addictions in DSM-5.^1^ This is the only behavioural addiction in this position, the argument being that only gambling disorder had the research evidence base supporting the transition.^2^

In Britain there is currently insufficient help for people with gambling problems and those affected by someone else's gambling such as family members.^3^ In this editorial we argue for an integration of gambling treatment service provision into existing drug and alcohol treatment services, and also for the commissioning of such services to be included within the local public health departments' remit, mirroring drug and alcohol treatment services. We believe that such a model will positively improve the status quo, wherein almost all of the gambling treatment services in Britain are funded by voluntary contributions from the gambling industry.

## Gambling as a public health problem: the scale

In Britain, 73% of adults gambled in the past 12 months,^4^ with the majority doing so without any harm to themselves or others. However, 0.9% (about 450 000 adults) gamble to a degree that damages or disrupts their personal, family or recreational pursuits, and such people are described as problem gamblers.^4^

The *British Gambling Prevalence Survey*^4^ found that 6.5% of the population were ‘at risk’ of becoming problem gamblers in the future. Especially vulnerable were Black and minority ethnic groups, young people and people with mental health and substance misuse problems. For those who gamble at risky levels, brief psychological interventions offered early on in their ‘gambling careers’ have been found to be effective in preventing the progression of their ‘at risk’ gambling to more problematic behaviours.^5,6^

Akin to other addictions, gambling disorder, if untreated, can result in a wide range of negative consequences to the individual and those around them. People addicted to gambling may commit crime to fund their addiction. As the addiction takes hold, employment and productivity may suffer significantly. Problem gamblers suffer from high rates of psychiatric comorbidity,^7^ and several stress-related and other medical disorders, with resultant increased utilisation of medical services.^8^ It has also been estimated that for every person addicted to gambling, up to eight others are also directly affected including family, friends and colleagues.^9^ Domestic violence and abuse are common,^10^ and children of gamblers have been found to have high rates of behavioural problems, emotional difficulties and substance misuse.^11^

## The policy context

Britain has liberal laws regulating gambling and this has resulted in some significant new trends in this field.^12^ First is the clustering of betting shops on the high streets. Second, the introduction of 33 000 fixed-odds betting terminals into betting shops across Britain.^13^ Third, a rapid expansion in remote gambling (i.e. internet and telephone betting), which is currently a fifth of the ‘offline’ gambling (where the gambler needs to by physically present) market.^14^ Fourth, a large increase in exposure to gambling advertising on television (the number of gambling advertising spots on television increased from 152 000 in 2006 to 1.39 million in 2012).^15^

## Current treatment and commissioning in Britain

For the nearly half a million problem gamblers and the 6.5% at risk, as well as those in their social networks who are affected, there is only one National Health Service (NHS) clinic and a patchy string of third-sector agencies that provide treatment. Some key limitations of the way current treatment providers operate include lack of a clearly defined model of care (such as a tiered care or a public health multi-tiered prevention approach as exists in the field of substance addictions), inadequate engagement with primary care or specialist mental health services, absence of regional commissioning protocols and lack of integrated care pathways.

None of the existing drug or alcohol treatment services offer fully integrated treatment for gamblers and their families, although some work in partnership with the charity GamCare (www.gamcare.org.uk) to provide some support for problem gamblers.

It is also important to note here that all funding for gambling treatment services (except Gamblers Anonymous and those in the private sector) comes exclusively from the gambling industry. An industry that generates billions of pounds in revenue every year, and growing year on year, voluntarily donates approximately 0.1% of that to fund gambling treatment, research and education in Britain. The Gambling Act 2005 enshrined the principle of ‘polluter pays’ regarding gambling treatment. Also, a mandatory levy on the gambling industry has often been proposed but has not been adopted as yet.

The commissioning of gambling treatment services is currently carried out by the Responsible Gambling Trust (www.responsiblegamblingtrust.org.uk), a charity. The Trust in turn works closely with the Responsible Gambling Strategy Board, an independent body that advises the Gambling Commission (and in turn the Department for Culture, Media and Sport) on gambling research, treatment and education-related issues. In terms of commissioning, the Responsible Gambling Trust does most of its work through GamCare, which in turn sub-contracts various treatment services across the country. The major limitation of this commissioning model is the lack of purchaser-provider split. In Britain, the government takes no responsibility for the commissioning of gambling treatment services, as strikingly made evident by the Department for Culture, Media and Sport having responsibility for gambling rather than the Department of Health.

## An integrated model of service provision

We call for gambling treatment service provision to be integrated within the existing network of drug and alcohol treatment services. These services are provided through a network of local treatment agencies (often partnerships of NHS and third-sector agencies) and are commissioned through public health departments of local councils. We feel this would be the most cost-efficient and sustainable way to deliver services and to enhance existing service provision. Major advantages of using existing drug and alcohol treatment services as a vehicle to deliver gambling treatment are the infrastructure and personnel cost efficiencies in terms of not generating separate set-up and running costs. We envisage some additional investment requirements primarily in terms of staff training and support to ensure their skill set broadens enough to include gambling treatment interventions. There might also be a need to increase personnel capacity, dependent on the volume of additional activity this generates. All this can only successfully happen if existing addiction treatment services are fully signed up to this, both strategically and operationally. This is not to say that specialist gambling treatment centres do not have a role to play: they could serve as centres of research and academic excellence, also leading on policy and strategy matters in a hub and spoke model, as described in the *Gambling: The Hidden Addiction* document.^12^

### Integrated commissioning of substance and gambling addiction treatment services

If gambling treatment provision were to be integrated into drug and alcohol services, it logically follows that commissioning of these services should also sit together. Since the recent shake-up in NHS commissioning processes, current commissioning of drug and alcohol services has become the responsibility of the local councils, through their public health departments. This commissioning structure provides a robust means to procure, monitor and manage the performance of treatment providers, and including gambling addictions services into this structure would be an improvement on the status quo. For this to happen, more collaborative work would be required between the Responsible Gambling Trust and local public health departments. It seems an opportune time to consider local pilot models of service delivery to test the feasibility and effectiveness of this proposal.

We acknowledge there is a lack of robust international evidence to support this model. However, we believe that Britain is in a unique position to be able to test it, as it allows for multiple sources of funding both from the existing Responsible Gambling Trust and from the NHS and Public Health England sources. This will help create a more robust support structure of treatment services and commissioning throughout the country, with a tiered approach, leaving the NHS services to deal with the more severe presentations either in terms of chronicity and severity of the illness or in terms of comorbid presentation.

Gambling disorder is now acknowledged as a valid psychiatric condition and as such, similar to other substance addictions. To integrate its treatment provision and commissioning into the existing infrastructure for drug and alcohol treatment and commissioning, treatment services, public health commissioners and other key stakeholders need to act in unison and without further delay. Not only will this rightly help raise the policy, research and practice profiles of gambling addiction, but it will also provide better care for gamblers and their families without significant cost increments.
